# Factors associated with acute kidney injury among preterm infants administered vancomycin: a retrospective cohort study

**DOI:** 10.1186/s12887-023-04085-z

**Published:** 2023-06-16

**Authors:** Baek Sup Shin, Seung Han Shin, Seul Gi Park, Ee-Kyung Kim, Han-Suk Kim

**Affiliations:** Department of Pediatrics, Seoul National University Children’s Hospital, Seoul National University College of Medicine, 101, Daehak-Ro, Jongno-Gu, Seoul, 03080 Republic of Korea

**Keywords:** Vancomycin, Piperacillin-Tazobactam, Preterm infant, Very low birth weight infant, Nephrotoxicity, Acute kidney injury

## Abstract

**Background:**

Vancomycin (VCM) is a widely used antibiotic for the treatment of gram-positive microorganisms, with some nephrotoxic effects. Recent studies have suggested that piperacillin-tazobactam (TZP) aggravates VCM-induced nephrotoxicity in adults and adolescents. However, there is a lack of research investigating these effects in the newborn population. Therefore, this study investigates whether the concomitant use of TZP with VCM use increases the risk of acute kidney injury (AKI) and to explore the factors associated with AKI in preterm infants treated with VCM.

**Methods:**

This retrospective study included preterm infants with birth weight < 1,500 g in a single tertiary center who were born between 2018 and 2021 and received VCM for a minimum of 3 days. AKI was defined as an increase in serum creatinine (SCr) of at least 0.3 mg/dL and an increase in SCr of at least 1.5 times baseline during and up to 1 week after discontinuation of VCM. The study population was categorized as those with or without concomitant use of TZP. Data on perinatal and postnatal factors associated with AKI were collected and analyzed.

**Results:**

Of the 70 infants, 17 died before 7 postnatal days or antecedent AKI and were excluded, while among the remaining participants, 25 received VCM with TZP (VCM + TZP) and 28 VCM without TZP (VCM—TZP). Gestational age (GA) at birth (26.4 ± 2.8 weeks vs. 26.5 ± 2.6 weeks, *p* = 0.859) and birthweight (750.4 ± 232.2 g vs. 838.1 ± 268.7 g, *p* = 0.212) were comparable between the two groups. There were no significant differences in the incidence of AKI between groups. Multivariate analysis showed that GA (adjusted OR: 0.58, 95% CI: 0.35–0.98, *p* = 0.042), patent ductus arteriosus (PDA) (adjusted OR: 5.23, 95% CI: 0.67–41.05, *p* = 0.115), and necrotizing enterocolitis (NEC) (adjusted OR: 37.65, 95% CI: 3.08–459.96, *p* = 0.005) were associated with AKI in the study population.

**Conclusions:**

In very low birthweight infants, concomitant use of TZP did not increase the risk of AKI during VCM administration. Instead, a lower GA, and NEC were associated with AKI in this population.

## Background

Vancomycin (VCM) is an antibiotic that is widely used for the treatment of infections caused by gram-positive microorganisms, including methicillin-resistant *Staphylococcus aureus* (MRSA) and coagulase-negative *Staphylococcus* species found in neonatal intensive care units (NICUs) [1]. However, nephrotoxicity is a well-known side effect of VCM that may lead to acute kidney injury (AKI) in preterm infants [2].

There have been few studies on the risk factors for VCM-induced AKI in the pediatric population, especially those examining higher doses of VCM or longer duration of treatment [3]. Other risk factors for AKI during VCM treatment include co-administration of nephrotoxic drugs and age of < 12 months [4–6]. In one propensity-matched cohort study of newborn infants, the presence of patent ductus arteriosus (PDA), concomitant use of non-steroidal anti-inflammatory drugs, bacteremia, and low birth weight are associated with VCM-induced AKI [7]. Additionally, higher trough levels, hypotension, and furosemide use have also been associated with AKI [8]. However, in a retrospective study of preterm infants, VCM-induced nephrotoxicity is rare, even with high peak serum concentrations [9].

Recently, concerns have been raised that VCM-induced nephrotoxicity may be aggravated by combination therapy with piperacillin-tazobactam (TZP) [10]. In a single-center retrospective study of an adult population, concurrent use of TZP and a higher trough concentration of VCM increased the incidence of nephrotoxicity [11]. Moreover, in a multicenter retrospective study of hospitalized children, co-administration of VCM and TZP increased the risk of AKI [12]. As piperacillin inhibits tubular secretory clearance, decreased clearance of VCM may play a role in the increased risk of AKI during co-administration of TZP [13]. However, there is a lack of data indicating that concomitant use of VCM and TZP increases the risk of AKI in the newborn population [14].

Therefore, this retrospective cohort study investigates whether the administration of TZP increases the risk of AKI during VCM use and explores factors associated with AKI during VCM treatment in preterm infants.

## Methods

This was a single-center, retrospective cohort study that compared the adverse effects of VCM administered either in combination TZP or without on renal function among very low birth weight infants (< 1,500 g) in NICUs between January 2018 and December 2021. Infants who had received VCM for at least three days were enrolled in the study, and those who died before seven postnatal days or had antecedent AKI were excluded from the study population. The participants’ medical records were reviewed, including birth weight, gestational age (GA), intrauterine growth restriction, oligohydramnios, delivery mode, sex, Apgar score at 1 and 5 min, duration of VCM treatment, concomitant use of other antibiotics, body weight at initial VCM administration, VCM serum concentration, pathogen-proven sepsis, creatinine level before and after VCM treatment, lowest mean blood pressure, inotropes, history of treated PDA, and necrotizing enterocolitis (NEC) at or before VCM treatment. NEC was defined according to the modified Bell’s criteria of grade 2 or more [15]. Baseline laboratory values of hemoglobin, albumin, and creatinine within 48 h before administration of antibiotics, and subsequent values of creatinine during and up to 1 week following VCM discontinuation, were collected. Initial therapeutic drug monitoring (TDM) was performed before the fourth dose of VCM, and follow-up TDMs were conducted when recommended by the Department of Laboratory Medicine.

All TDMs were collected at the trough, 30 min prior to VCM administration. VCM at a concentration of > 40 mg/mL was considered high, based on previous studies of VCM-induced nephrotoxicity [16–18].

The study population was first divided into the VCM + TZP group and the VCM–TZP group according to the concomitant use of TZP. Those who received VCM only as monotherapy and those who received antibiotics other than TZP were categorized as the VCM—TZP group. AKI was defined as an increase in serum creatinine (SCr) of at least 0.3 mg/dL within 48 h or an increase in SCr of at least 1.5 times the baseline level, as recommended by the latest version of Kidney Disease: Improving Global Outcomes (KDIGO) clinical practice guidelines for AKI published in 2012 [19]. Baseline creatinine was defined as the last creatinine level prior to VCM administration, as creatinine was measured weekly in the study population. SCr within 1 week preceding VCM treatment and the highest SCr during and 1 week following VCM discontinuation were measured. The study population was then re-categorized into AKI and non-AKI groups to investigate risk factors for this condition.

Data analysis was performed using R version 4.1.3 (R Foundation for Statistical Computing, Vienna, Austria). Continuous variables were analyzed using the Student’s T test, Welch’s T test, or Wilcoxon rank sum test. The proportions were analyzed using Fisher’s exact test. Single-variable and multivariable logistic regression analyses were used to assess the risk factors for AKI. Variables with a P-value < 0.1 in the univariate analysis were included in the multivariable analysis. Statistical significance was set at P < 0.05. If normality and homoscedasticity was satisfied, data were presented as mean ± standard deviation (SD). Otherwise, data were presented as median [interquartile range], and categorical variables were presented as rate.

## Results

**Fig. 1:**
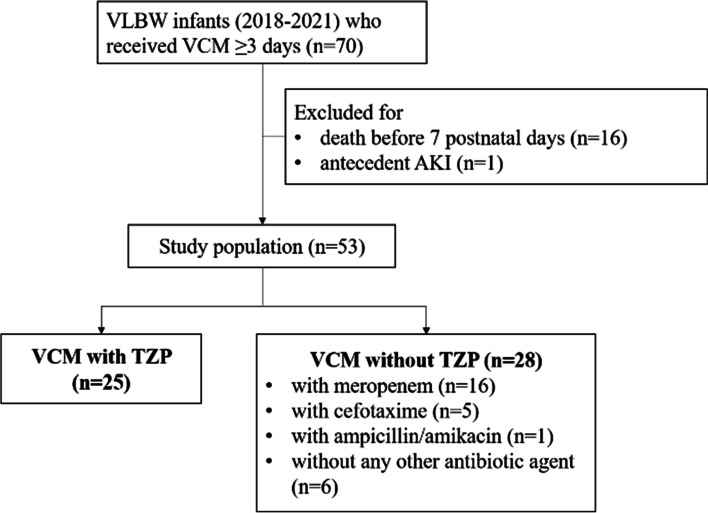
Patient selection diagram. VLBW, very low birthweight; AKI, acute kidney injury; VCM, vancomycin; TZP, piperacillin/tazobactam

Of the 70 infants who received VCM, 16 who died before 7 days of life and one who experienced antecedent AKI were excluded. Among the 53 infants ultimately analyzed, 25 received VCM with TZP (VCM + TZP), and the remaining 28 did not receive TZP (VCM—TZP). Of the 28 VCM—TZP infants, 16 received meropenem concomitantly, five received cefotaxime, one received ampicillin and amikacin, and the remaining six received only VCM as monotherapy (Fig. 1).

There were no differences in birth weight (750.4 ± 232.2 g vs. 838.1 ± 268.7 g, *p* = 0.212) or gestational age at birth (26.4 ± 2.8 weeks vs. 26.5 ± 2.6 weeks, *p* = 0.859) between the VCM + TZP and VCM—TZP groups (Table 1). Similarly, no differences were found in the other perinatal characteristics between the two groups. There were also no differences in use of inotropic agents and ibuprofen during VCM treatment, lowest blood pressure during VCM treatment, proven sepsis, or NEC before or after VCM treatment between the two groups.

**Table 1:** Baseline characteristics of the study population

	VCM + TZP	VCM—TZP	*P* value
	(*N* = 25)	(*N* = 28)	
Gestational age, week	26.4 ± 2.8	26.5 ± 2.6	0.859
Birth weight, g	750.4 ± 232.2	838.1 ± 268.7	0.212
Oligohydramnios	3 (13.6)	4 (16.0)	1.000
Male	14 (56.0)	16 (57.1)	1.000
IUGR	7 (28.0)	4 (14.3)	0.374
Apgar score, 1 min	3.8 ± 1.7	3.1 ± 2.0	0.216
Apgar score, 5 min	6.6 ± 2.1	5.9 ± 2.3	0.278
Hemoglobin	9.7 [9.2–10.8]	11.1 [9.4–12.4]	0.090
Baseline creatinine	0.5 [0.4–0.9]	0.5 [0.3–0.7]	0.605
Albumin	3.0 ± 0.5	3.1 ± 0.4	0.475
Duration of receiving vancomycin, days	4.0 [2.0–15.0]	3.5 [2.5–5.0]	0.401
PMA at starting vancomycin, week	33.8 ± 7.5	35.0 ± 9.0	0.580
Weight at starting vancomycin, kg	1,090 [680–2,100]	1,490 [755–2,710]	0.265
Pathogen proven sepsis	10 (40.0)	11 (39.3)	1.000
Lowest mean blood pressure, mmHg	36.6 ± 11.7	37.1 ± 9.5	0.843
Use of Inotropes	10 (40.0)	8 (28.6)	0.558
PDA	22 (88.0)	24 (85.7)	1.000
PDA at starting vancomycin	12 (48.0)	13 (46.4)	1.000
Use of ibuprofen	11 (44.0)	18 (64.3)	0.228
Use of ibuprofen at starting vancomycin	5 (20.0)	6 (21.4)	1.000
PDA ligation operation	11 (44.0)	9 (32.1)	0.545
NEC	4 (16.0)	3 (10.7)	0.872
IVIG during vancomycin	10 (40.0)	6 (21.4)	0.242

Data are presented as mean ± SD, median [interquartile range], or n (%)

*IUGR* Intrauterine growth restriction, *PMA* postmenstrual age, *PDA* Patent ductus arteriosus, *NEC* Necrotizing enterocolitis, *IVIG* Intravenous immunoglobulin

Additionally, there was no difference in the incidence of AKI between the two groups (21.4% vs. 16.0%, *p* = 0.732) (Table 2). Increase in SCr during or after VCM treatment (0.5 ± 0.6 vs. 0.5 ± 0.6 mg/dL, *p* = 0.766) and the ratio of SCr before and after VCM treatment (1.2 ± 0.9, vs. 1.3 ± 0.5, *p* = 0.649) were comparable between the two groups.

**Table 2:** Creatinine level, acute kidney injury, and serum concentration of vancomycin

	VCM + TZP (*N* = 25)	VCM—TZP (*N* = 28)	*P* value
Difference of creatinine, mg/dL	0.5 ± 0.6	0.5 ± 0.6	0.766
Ratio of creatinine	1.2 ± 0.9	1.3 ± 0.5	0.649
Occurrence of AKI	4 (16.0)	6 (21.4)	0.732
Highest concentration of VCM, μg/mL	37.4 [27.6–48.5]	35.9 [29.7–40.6]	0.745
Lowest concentration of VCM, μg/mL	13.8 [ 8.2–20.9]	12.0 [10.0–18.1]	0.882

Data are presented as mean ± SD, median [interquartile range], or n (%). Difference and ratio of creatinine were analyzed using student T test and occurrence of AKI was analyzed using Fisher’s exact test. The data of VCM concentrations of 19 infants in the VCM + TZP group and 21 in the VCM—TZP group were available

*VCM* vancomycin, *TZP* piperacillin/tazobactam, *AKI* acute kidney injury

TDM of the VCM was conducted in 19 infants in the VCM + TZP group and 21 in the VCM—TZP group. The highest and lowest concentrations of VCM and incidence of high VCM levels (> 40ug/mL) were comparable between the TZM + VCM and TZM—VCM groups.

Patients receiving VCM were also grouped into AKI and non-AKI groups to explore risk factors for VCM-induced AKI. In the study population, the overall incidence of VCM-induced AKI was 18.9%. GA at birth and history of NEC were significantly different between the two groups (Table 3). In addition, backward stepwise regression showed that low GA (adjusted OR: 0.58, 95% CI: 0.35–0.98) and a previous history of NEC (37.65, 3.08–459.96) were associated with AKI.

**Table 3:** Univariable and multivariable analyses for acute kidney injury

	AKI (*N* = 10)	non-AKI (*N* = 43)	Univariate OR (95% CI)	*P*-value	^a^Multivariate OR (95% CI)	*P*-value
Gestational age, week	24.6 [24.3–25.6]	26.4 [24.4–28.4]	0.63 (0.39–1)	0.052	0.58 (0.35–0.98)	0.042
IUGR	1 (10.0)	10 (23.3)	0.37 (0.04–3.25)	0.368		
Duration of VCM, days	5.0 [3.0–9.0]	4.0 [2.0–9.0]	1.02 (0.94–1.10)	0.701	-	-
PMA at starting VCM, week	27.8 [25.1–40.4]	33.0 [29.2–38.7]	0.96 (0.87–1.05)	0.356	-	-
Highest concentration of VCM, μg/mL	38.5 [29.8–60.5]	36.2 [25.1–44.1]	1.04 (0.99–1.10)	0.088	-	-
PDA at starting VCM	7 (70.0)	18 (41.9)	3.24 (0.74–14.26)	0.12	5.23 (0.67–41.05)	0.115
NEC	5 (50.0)	2 (4.7)	20.05 (3.11–134.94)	0.002	37.65 (3.08–459.96)	0.005

Data are presented as median [interquartile range], or n (%)

*IUGR* Intrauterine growth restriction, *PMA* postmenstrual age, *PDA* patent ductus arteriosus, *NEC* necrotizing enterocolitis, *AKI* acute kidney injury, *OR* odds ratio, *CI* confidence interval

^a^Adjusted for gestational age and PDA at initiation of VCM and NEC

## Discussion

This study found no difference in the incidence of AKI among very low birthweight infants who were treated with VCM and TZP and those treated with VCM and no TZP, which is in agreement with a previous study by Bartlett et al. [14]. Further, the overall incidence of VCM-associated AKI was 18.9% in the study population. We instead found that gestational age at birth and preceding NEC episodes were associated with AKI during VCM treatment.

One possible reason that this study’s results differed from those that examined the same treatments of a pediatric population and found TZP to have additive effect on VCM-induced AKI might have been the characteristics of the study population. In a previous study of the pediatric population, the incidence of AKI was 11.7% in the VCM with TZP group, while only 4% in VCM with the cefepime group [12]. Moreover, a single-center retrospective study reported the rate of VCM-associated AKI from the concomitant use of TZP in pediatric patients to be 28.9%, compared with those without TZP in 7.9% [20]. Additionally, another retrospective study that demonstrated the use of TZP as a risk factor for VCM-associated AKI in children showed that the incidence of AKI was 12.6% in the study population [4]. However, in the present study, the incidence of AKI was as high as 19%, indicating a higher prevalence of AKI than that in the pediatric population.

Notably, one systematic review suggesting that concomitant use of VCM and TZP was associated with AKI in the pediatric population also reported that subgroup analysis of the study population in the intensive care unit showed that more than 50% showed no increase in AKI risk [21]. AKI is also prevalent in NICU, as a multicenter, multinational, observational cohort study reported incidence of AKI as high as 30% in patients [22].

Although precedent AKI was excluded from the study population in this study, precedent NEC was associated with AKI, and NEC was common in preterm infants who developed NEC [23]. In a previous study, half of the participants received ibuprofen, and one out of five infants did so at the time of VCM treatment. Non-steroidal anti-inflammatory drugs are the most common medications that induce nephrotoxicity next to antibiotics in preterm infants [24].

A high incidence of AKI in a study population may attenuate the impact of other known factors on AKI, such as the serum concentration of VCM [8]. In this study, GA, the most profound factor associated with various neonatal morbidities in preterm infants, was associated with AKI in the study population instead of concomitant VCM/TZP treatment.

This study has some limitations, including its small sample size and retrospective design. Moreover, the association between AKI and VCM concentration could be underestimated, as there were missing values in the VCM concentration data for 13 patients from the non-AKI group. Also, potential effects of antibiotics other than VCM on the renal function were not fully demonstrated in this study, as relatively small number for patients received each agent. This can lead to bias, as a relatively high number of patients in the VCM—TZP group received different antibiotics other than VCM. However, an association between each of these drugs and AKI was not observed in the study population (data not shown). Preterm infants requiring VCM are usually systemically ill from serious infection or NEC. Although various factors that may influence renal function were reviewed in this study, preterm infants who require VCM also receive various medications and often exhibit decreased organ perfusion. Moreover, those who died or were excluded from the study population had the highest risk of kidney injury.

However, this study showed results consistent with a previous study showing that TZP did not increase the risk of AKI during VCM use [14].

## Conclusion

In this study, the concomitant use of TZP did not increase risk of AKI during VCM administration. However, lower GA and antecedent NEC were shown to be associated with AKI in this population.

## Data Availability

The datasets generated and analyzed are not publicly available but are available from the corresponding author upon reasonable request.

## Abbreviations

VCM: Vancomycin
TZP: Piperacillin-tazobactam
AKI: Acute kidney injury
VLBW: Very low birth weight
SCr: Serum creatinine
GA: Gestational age
OR: Odds ratio
CI: Confidence interval
aOR: Adjusted odds ratio
PDA: Patent ductus arteriosus
NEC: Necrotizing enterocolitis
MRSA: Methicillin-resistant *Staphylococcus aureus*
TDM: Therapeutic drug monitoring
KDIGO: Kidney Disease: Improving Global Outcomes
IVIG: Intravascular immunoglobulin
PMA: Postmenstrual age
